# Determination of nasal and oropharyngeal microbiomes in a multicenter population-based study – findings from Pretest 1 of the German National Cohort

**DOI:** 10.1038/s41598-017-01212-6

**Published:** 2017-05-12

**Authors:** Manas K. Akmatov, Nadine Koch, Marius Vital, Wolfgang Ahrens, Dieter Flesch-Janys, Julia Fricke, Anja Gatzemeier, Halina Greiser, Kathrin Günther, Thomas Illig, Rudolf Kaaks, Bastian Krone, Andrea Kühn, Jakob Linseisen, Christine Meisinger, Karin Michels, Susanne Moebus, Alexandra Nieters, Nadia Obi, Anja Schultze, Julia Six-Merker, Dietmar H. Pieper, Frank Pessler

**Affiliations:** 1 TWINCORE, Center for Experimental and Clinical Infection Research, Hannover, Germany; 2 Helmholtz Centre for Infection Research, Braunschweig, Germany; 3 Centre for Individualised Infection Medicine, Hannover, Germany; 4 Microbial Interactions and Processes, Helmholtz Centre for Infection Research, Braunschweig, Germany; 5 Leibniz Institute for Prevention Research and Epidemiology-BIPS, Bremen, Germany; 6 University Cancer Center Hamburg, University Medical Center Hamburg-Eppendorf, Hamburg, Germany; 7 Division of Cancer Epidemiology, German Cancer Research Centre, Heidelberg, Germany; 8 Institute for Social Medicine, Epidemiology and Health Economics, Charité-Universitätsmedizin Berlin, Berlin, Germany; 9 Department of Epidemiology, Helmholtz Centre for Infection Research, Braunschweig, Germany; 10 Institute of Molecular Epidemiology, Helmholtz Centre Munich, Munich, Germany; 11 Institute for Medical Informatics, Biometry and Epidemiology, University Hospital of Essen, Essen, Germany; 12 Institute of Epidemiology 2, Helmholtz Centre Munich, Munich, Germany; 13 Klinikum Augsburg, KORA and NAKO Study Center, Augsburg, Germany; 14 Institute for Prevention and Cancer Epidemiology, University Medical Center Freiburg, Freiburg, Germany; 15 Centre for Chronic Immunodeficiency, University Medical Center Freiburg, Freiburg, Germany

## Abstract

We examined acceptability, preference and feasibility of collecting nasal and oropharyngeal swabs, followed by microbiome analysis, in a population-based study with 524 participants. Anterior nasal and oropharyngeal swabs were collected by certified personnel. In addition, participants self-collected nasal swabs at home four weeks later. Four swab types were compared regarding (1) participants’ satisfaction and acceptance and (2) detection of microbial community structures based on deep sequencing of the 16 S rRNA gene V1–V2 variable regions. All swabbing methods were highly accepted. Microbial community structure analysis revealed 846 phylotypes, 46 of which were unique to oropharynx and 164 unique to nares. The calcium alginate tipped swab was found unsuitable for microbiome determinations. Among the remaining three swab types, there were no differences in oropharyngeal microbiomes detected and only marginal differences in nasal microbiomes. Microbial community structures did not differ between staff-collected and self-collected nasal swabs. These results suggest (1) that nasal and oropharyngeal swabbing are highly feasible methods for human population-based studies that include the characterization of microbial community structures in these important ecological niches, and (2) that self-collection of nasal swabs at home can be used to reduce cost and resources needed, particularly when serial measurements are to be taken.

## Introduction

Due to achievements in molecular and genetic techniques, biological specimens have been collected in epidemiological studies with increasing frequency during recent years^1^. Nasal and oropharyngeal swabs are required to examine microbial populations and dynamics of multidrug resistant pathogens (e.g. methicillin-resistant *Staphylococcus aureus*)^2–4^. However, technical and logistic difficulties may impede collection of such specimens, in particular when applied in population-based studies, as there is no immediately apparent benefit for participating in such studies such as receiving a medical diagnosis or detailed health care advice. Moreover, collecting specimens such as oropharyngeal swabs may be less acceptable due to physical discomfort and may thus decrease compliance and retention rates in prospective population-based studies.

The German National Cohort (GNC), in German referred to as the NaKo Gesundheitsstudie (http://nako.de), is a large population-based prospective study anticipating to recruit about 200.000 participants between 20 and 69 years of age across 18 study centers, with recruitment for the main study starting in late 2014^5^. The major aims of the GNC are to identify risk factors, including lifestyle-related, psychosocial, occupational, and environmental factors, for common diseases such as cardiovascular diseases, diabetes, cancer, and neuro-psychiatric, infectious, and musculoskeletal diseases. Participants’ microflora will be studied both as exposure variable that might affect the risk of common diseases, as an outcome variable modulated by the variables assessed (e.g., nutrition, lifestyle, common diseases), and to study dynamics of colonizing microbial populations and selected pathogens in this large human population over time. Considering the critical importance of accurate determination of microbiomes to address central questions of the GNC, we therefore conducted a feasibility study in Pretest 1 of the GNC, featuring 524 individuals recruited from the general population. The major aims were to (1) test the feasibility (including participants’ acceptance and satisfaction) of collecting nasal and oropharyngeal swabs in this population-based setting, (2) compare four swab types in the ability to detect microbial community structures at these two anatomical sites, and (3) to test whether equivalent microbial communities could be detected by nasal swabs collected by the participants at home compared to nasal swabs collected by trained personnel in the study center.

## Results

**Figure 1:**
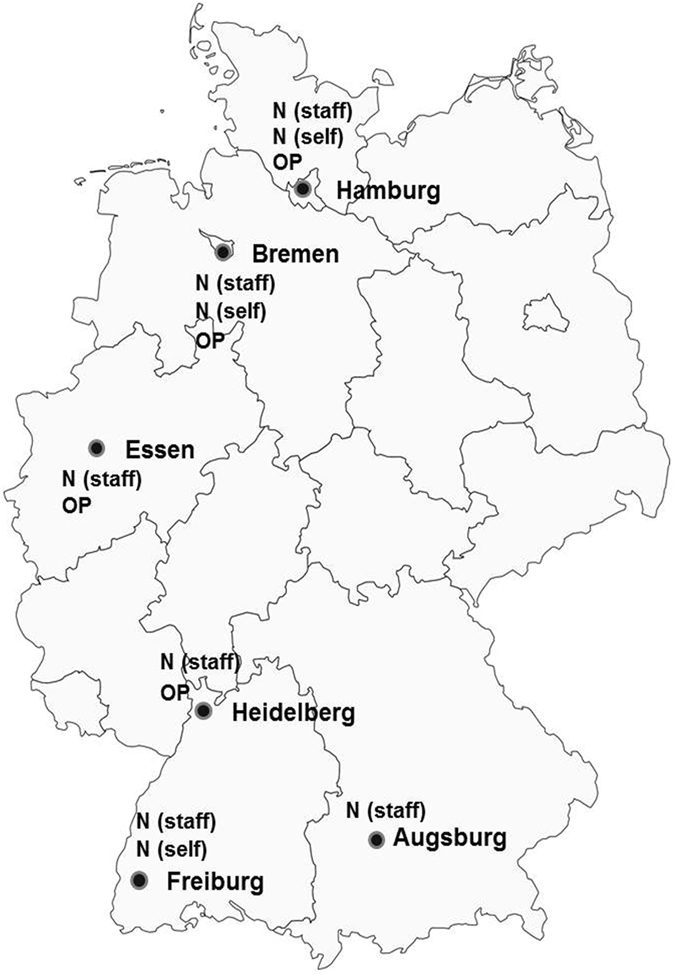
Overview of the participating study centers and swabbing methods applied. Abbreviations used: N (staff) = nasal swabs collected by certified personnel in the study center; N (self) = nasal swabs self-collected by the participants at home; OP = oropharyngeal swabs collected by certified personnel in the study center. The map was taken from: http://commons.wikimedia.org/wiki/Image:Karte_Bundesrepublik_Deutschland.svg and edited by the author (MKA). Name of the creator of the source map is David Liuzzo. Link to the license: https://commons.wikimedia.org/wiki/Commons:Reusing_content_outside_Wikimedia. The map was published under the CC Creative Commons Attribution-ShareAlike 2.5 Generic (CC BY-SA 2.5) license, which allows (https://creativecommons.org/licenses/by-sa/2.5/deed.en): • Share - copy and redistribute the material in any medium or format • Adapt - remix, transform, and build upon the material for any purpose, even commercially. • The licensor cannot revoke these freedoms as long as you follow the license terms.

Figure 1 shows the geographical locations of the six study centers and the type of swabbing conducted at each center.

### Preanalytical feasibility

#### Nasal swabbing at the study center

Nasal swabs were collected from nearly all participants in the study centers. Nasal swabs could not be collected from two participants: one participant was deemed mentally not competent to understand instructions and purpose of the study (Bremen), whereas no reason was given for the other participant (Freiburg). Complete sets of swabs were collected at the other study centers (Table 1, third column). The median time for nasal swabbing ranged from one to three minutes. In general, only few difficulties were reported by the study personnel (Table 1, fifth column). One participant was reported to have “dry mucous membranes” and being “anxious”. Swabs from both nostrils could be obtained from all participants except from three: in one case one nostril could not be swabbed because of the presence of nose piercing; a reason was not reported in the other two cases. A small fraction of participants reported the urge to sneeze and/or sneezing (20%), fewer reported tearing (5.5%); only a few reported pain (0.91%).

**Table 1:** Selected feasibility aspects of nasal and oropharyngeal swabbing.

Column 1	2	3	4	5	6	7	8	9	10	11	12	13	14	15	16
	Nasal swabs collected by study personnel at the study center						Oropharyngeal swabs collected by study personnel at the study center					Nasal swabs collected by study participants at home			
Study centers	Sample size *n*	No. of partici-pants from whom swabs were collected *n*	Time needed for swabbing in minutes *median (range)*	Difficul-ties when collecting the swab *% of “No” responses*	Swabs from both nares were taken *% of “Yes” responses*	Swabs that had to be discarded *% of „None“ responses*	Sample size *n*	No. of partici-pants from whom swabs were collected *n (%)*	No. of partici-pants who refused oropha-ryngeal swabbing *n (%)*	Time needed for swabbing in minutes *median (range)*	Difficul-ties when collecting the swab *% of “Yes” responses (n/N)*	No. of partici-pants to whom swabs were sent *n*	No. of partici-pants from whom swabs were received *n (%)*	Time from mailing of the swab kit to swabbing (days) *Median (range)*	Time from swabbing to swab receipt in the lab (days) *Median (range)*
Augsburg	100	100	1 (<1–6)	100	100	100	—	—	—	—	—	—	—	—	—
Bremen	96	95	3 (1–10)	98	100	100	95	81 (85)	14 (15)	2 (<1–5)	68 (54/79)	94	83 (88)	8 (2–63)	1 (1–13)
Essen	75	75	3 (<1–7)	95	100	100	75	73 (97)	2 (2.7)	2 (1–4)	42 (29/69)	—	—	—	—
Freiburg	97	96	2 (1–5)	98	98	100	—	—	—	—	—	98	69 (70)	11 (4–44)	1 (1–30)
Hamburg	100	100	2 (<1–5)	98	99	100	100	99 (99)	1 (1.0)	1 (<1–12)	53 (52/99)	100	75 (75)	8 (2–66)	2 (1–30)
Heidelberg	56	56	2 (<1–5)	100	100	98	56	56 (100)	0 (0)	1 (<1–6)	36 (20/56)	—	—	—	—

#### Oropharyngeal swabbing at the study center

**Figure 2:**
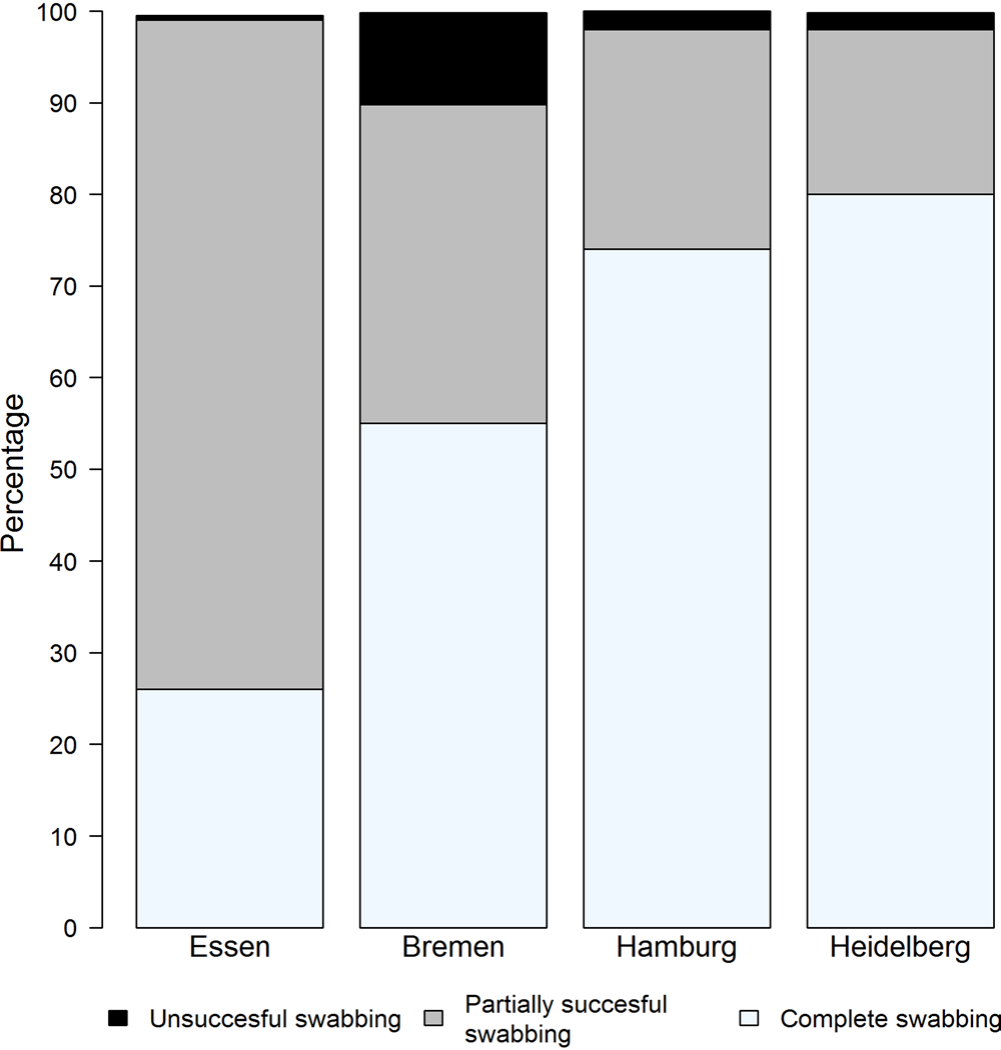
Completeness of oropharyngeal swabbing. The fraction of the anatomical structures swabbed successfully was recorded by the certified study personnel in the biosample protocol. Complete swabbing – all target structures (tonsils and pharyngopalatine arches bilaterally) could be swabbed; partially successful swabbing – the swab touched the mucous membrane of the pharynx in only one spot or only swabbed some areas of the pharynx; unsuccessful swabbing - the swab did not touch the oropharyngeal mucous membrane; i.e., the swab could not be collected.

Between ~85% and 100% of study participants agreed to oropharyngeal swabbing (Table 1, ninth column). Those who refused oropharyngeal swabbing reported “unpleasant reaction to oropharyngeal swabbing in the past” as a reason for refusal. The median time needed for oropharyngeal swabbing ranged, by study center, from one to two minutes (Table 1, 11^th^ column). Problems or difficulties were reported in about half of the oropharyngeal swabs (155/303). The percentage of problems or difficulties reported by the study personnel varied between ~36% and ~68%. “Retching” was the main problem reported by the participants in all recruitment centers (97%, 149/155), but frank vomiting was not reported. “Defense reaction” was reported in 5.8% (9/155, multiple choice was possible). The completeness of oropharyngeal swabbing differed by recruitment center (Fig. 2); all target structures of the pharynx could be swabbed in the majority of cases in two centers, but in only about 20% in one of the others.

#### Nasal self-swabbing at home

The percentage of nasal swabs that were returned to the study center ranged from 70% to 88% (Table 1, 14^th^ column). In the three study centers participating in this arm of the study, the median time from mailing of the swab kit to the participants to self-swabbing was 9.5 days (range, 2 to 66 days). The median time from self-swabbing to receipt of the swab at the laboratory was 2 days (range, 1 to 30 days). The study participants reported only minor discomfort such as tearing or sneezing when self-collecting the nasal swab. Pain at swabbing was reported in only two cases.

### Acceptance and preference

**Figure 3:**
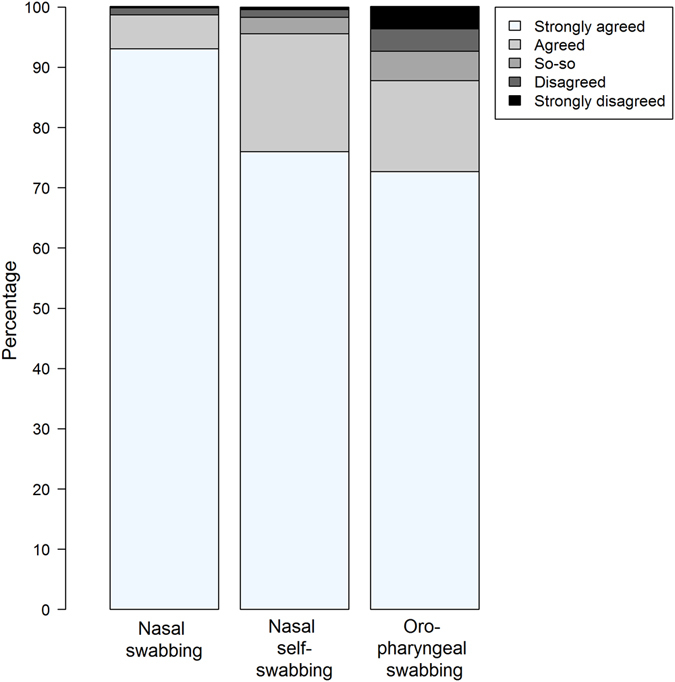
Acceptance of nasal and oropharyngeal swabbing. Responses to the following 3 statements were evaluated using the Likert scale shown in the legend: (1) Collection of the nasal swabs by the study personnel was acceptable to me; (2) Collecting the nasal swab at home was acceptable to me; (3) Collection of the throat swab by the study personnel was acceptable to me.

All swabbing methods were highly accepted by the study participants (Fig. 3). There were only minor differences in acceptance and preference across study centers (Table 2). A considerable proportion of participants reported that they would participate in future studies in which collection of nasal or oropharyngeal swabs is planned. Participants had no preference regarding who should collect the nasal swab (either study personnel or the participant him/herself). Compared to nasal swabs, participants reported less comfort when oropharyngeal swabs were collected (Table 1). The participants did not report technical difficulties when self-collecting nasal swabs.

**Table 2:** Participants’ acceptance of nasal and oropharyngeal swabbing at the study center (median and range).

Items	Augsburg n = 100	Bremen n = 91	Essen n = 37	Freiburg n = 94	Hamburg n = 49	Heidelberg n = 52
*Nasal swab collected by study personnel*						
I felt comfortable when the study personnel collected the nasal swab	5 (1–5)	5 (1–5)	5 (1–5)	5 (1–5)	5 (1–5)	5 (1–5)
I would participate again in a study in which a staff member collects a nasal swab from me	5 (1–5)	5 (1–5)	5 (1–5)	5 (1–5)	5 (1–5)	5 (2–5)
*Oropharyngeal swabs collected by study personnel*						
I felt comfortable when the study personnel collected the oropharyngeal swab	—	4 (2–5)	4.5 (1–5)	—	4.5 (1–5)	5 (1–5)
I would participate again in a study in which a staff member collects an oropharyngeal swab from me	—	5 (1–5)	5 (1–5)	—	5 (1–5)	5 (1–5)
*Nasal swabbing collected by the study participants*						
I felt comfortable when I collected the self-swab at home	—	5 (1–5)	—	5 (2–5)	5 (1–5)	—
I would rather conduct a nasal self-swab by myself than having it taken by study personnel	—	4 (1–5)	—	3 (1–5)	3 (1–5)	—
It was easy to collect the nasal self-swab	—	5 (1–5)	—	5 (3–5)	5 (1–5)	—
The instructions how to collect the self-swab were easy to understand	—	4 (1–5)	—	5 (1–5)	4 (1–5)	—
I would participate again in a study in which I self-collect a nasal swab	—	5 (1–5)	—	5 (2–5)	5 (1–5)	—

A 5-point Likert scale was used; 1 = strong disagreement, 2 = disagreement, 3 = neither agreement nor disagreement, 4 = agreement, and 5 = strong agreement.

### Analytical phase

Of the four swab types to be compared, swab 4 (a calcium alginate tipped swab) had to be excluded because the stabilizing gel interfered with cell lysis and thus caused DNA extraction problems. The following data were therefore obtained with swab types 1, 2 and 3.

#### Comparison of anterior nasal vs. oropharyngeal microbial communities

**Figure 4:**
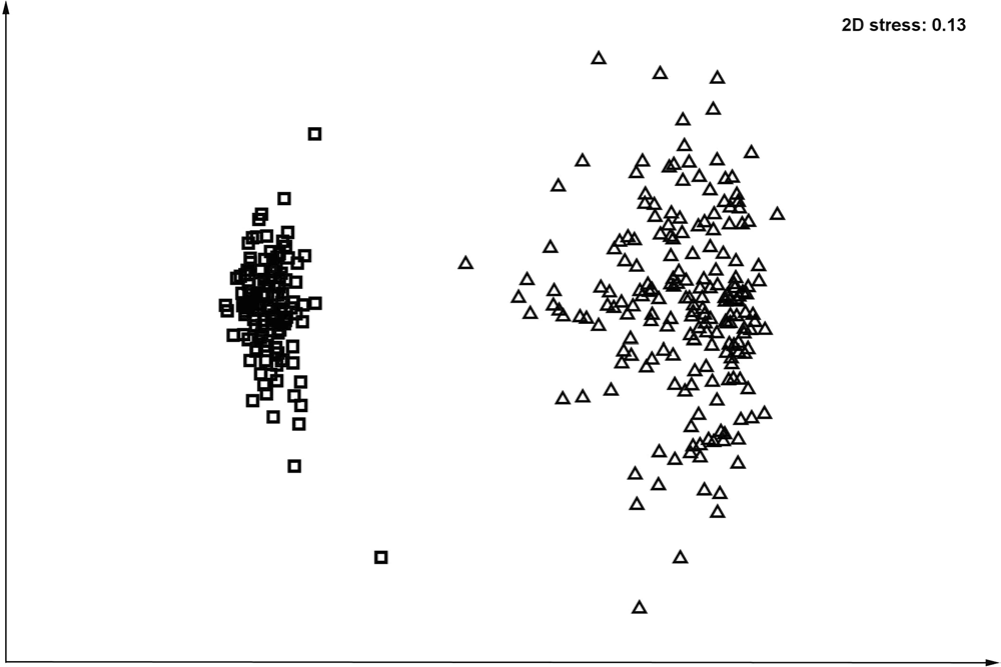
Global differences between anterior nasal and oropharyngeal microbial communities. Non-metric multidimensional scaling (nMDS) plot of the global bacterial community structure of 195 human anterior nasal (triangles) and 118 human oropharyngeal (squares) samples. The phylotype abundances (% sequence reads) were standardized but not transformed prior to the use of the Bray-Curtis similarity algorithm.

The microbial community structure analysis of the two tested ecological niches, the human oropharynx and the human anterior nares, revealed the presence of 846 phylotypes (PT) (see supplementary Table 1), 46 of which were exclusively found in oropharyngeal communities and 164 exclusively in nasal communities (see supplementary Table 2). The communities of these two habitats differed drastically, forming two separate clusters in the non-metric multidimensional scaling (nMDS) plot (Fig. 4). The oropharyngeal communities tended to cluster more tightly together than the nasal communities; in agreement with, multivariate dispersion analysis showed that the index of dispersion was higher for anterior nasal communities (1.09) than for oropharyngeal communities (0.73). This is in accordance with the higher average similarity between all pairs of oropharyngeal communities of 34.8%, compared to only 26.5% between all pairs of anterior nasal communities.

The anterior nasal communities were dominated by Actinobacteria (mean abundance of 45% in a total of 195 samples analyzed), Firmicutes (40%) and Proteobacteria (12%), along with minor amounts of Bacteroidetes (2%). Consistent with a previous report by Camarinha-Silva *et al*.^6^, the most abundant species were *Corynebacterium accolens/segmentosum* (PT1, 17%), *Propionibacterium acnes* (PT2, 12%), *Staphylococcus epidermidis* (PT3, 10%), and *Staphylococcus aureus* (PT5, 8%), which sum up to an average abundance of 47%.

In contrast, the oropharyngeal communities were not dominated by just a few species: the most abundant phylotypes (PT15, *Leptotrichia* sp.; PT12, *Fusobacterium periodonticum*; PT16, *Streptococcus salivarius/vestibularis*; PT19, *Veillonella atypical*; PT20, *Prevotella melaninogenica;* and PT21, *Prevotella histicola*) were observed at an average abundance of only 3.3–5.1%. Members of five phyla were abundant in the oropharyngeal communities (38% Firmicutes, 22% Bacteroidetes, 18% Fusobacteria, 11% Actinobacteria and 10% Proteobacteria). Thus, the overall microbial community observed here resembles that reported previously for oropharyngeal swabs ^7^. Notably, the oropharyngeal communities were richer in species (average number of phylotypes per sample: 201 ± 41) than the anterior nasal communities (87 ± 37 phylotypes per sample). They also showed higher species diversity using both Shannon and Simpson Diversity Indices (H′ = 3.86 ± 0.33 for oropharyngeal vs. 2.17 ± 0.60 for nasal communities and 1-D = 0.96 ± 0.02 for oropharyngeal vs. 0.76 ± 0.14 for nasal communities) but higher species evenness (J′ = 0.73 ± 0.04 for oropharyngeal vs. 0.49 ± 0.11 for nasal communities).

#### Comparison of swab types 1, 2 and 3

**Figure 5:**
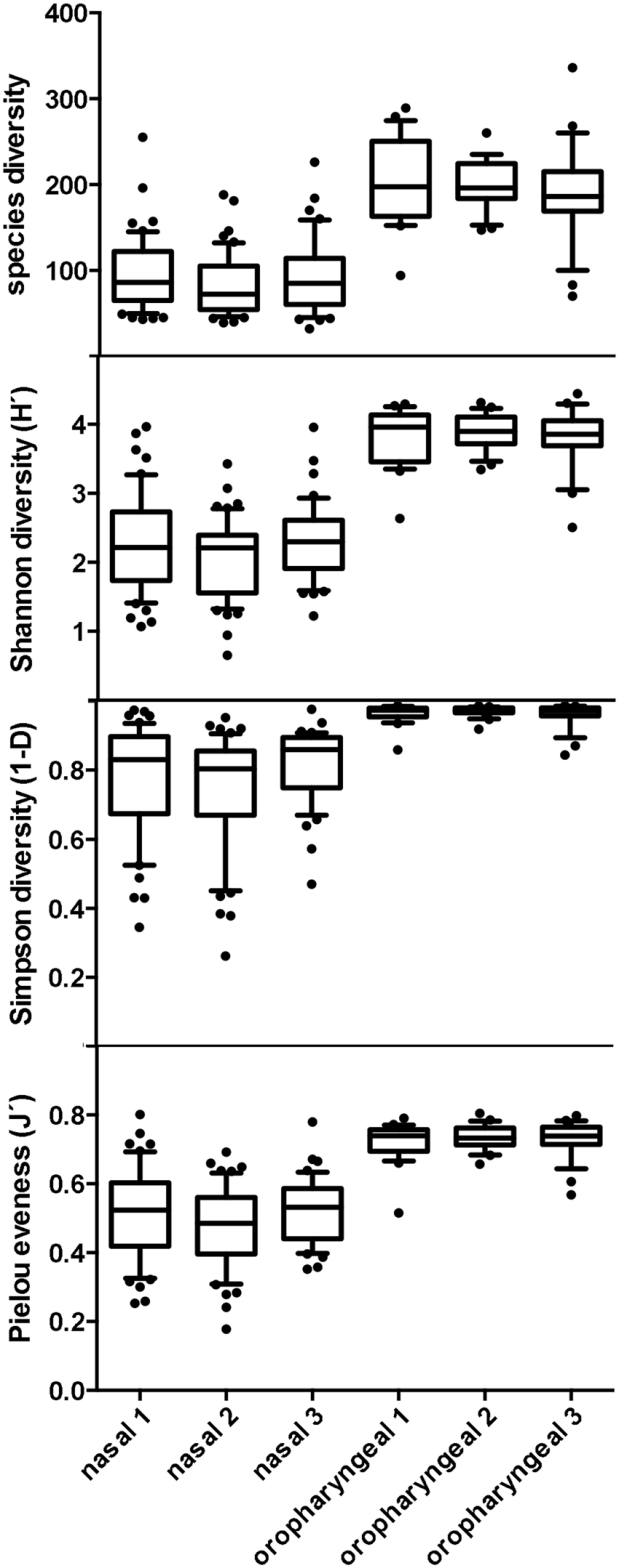
Community diversity in anterior nares and oropharynx according to swab type. The species richness, Shannon diversity index (H′), Simpsons diversity index (1-D), and Pielou’s evenness index of communities collected by three different swabs (swab 1, flocked nylon swab; swab 2, rayon swab; swab 3, polyurethane tipped swab) are shown. The whiskers of the plots indicate 10–90 percentiles. The number of swabs analyzed were 54 (swab 1), 52 (swab 2) and 44 (swab 3) nasal swabs and 39 (swab 1), 34 (swab 2) and 37 (swab 3) oropharyngeal swabs. There was no statistically significant difference in the diversity observed in communities obtained with the different swabs, whereas communities taken from the nares or the oropharynx were significantly different (p < 0.0001).

The analysis of diversity indices did not suggest any differences in microbial communities when sampling had been performed by each of the three swab types included in this analysis (Fig. 5). For instance, Simpson’s Diversity Index of oropharyngeal communities was close to 1 for all three swab types; nasal communities had significantly lower diversity indices but, again, there were no differences across swab types. Similarly, Analysis of Similarity (ANOSIM) and Permutational Multivariate Analysis of Variation (PERMANOVA) gave no evidence for overall differences in the communities detected by the different swab types (ANOSIM: global R = 0.001 for nasal and 0.015 for oropharyngal communities while PERMANOVA: Pseudo-F = 1.4522, p = 0.131 for nasal and Pseudo-F = 1.4383, p = 0.129 for oropharyngal communities).

**Figure 6:**
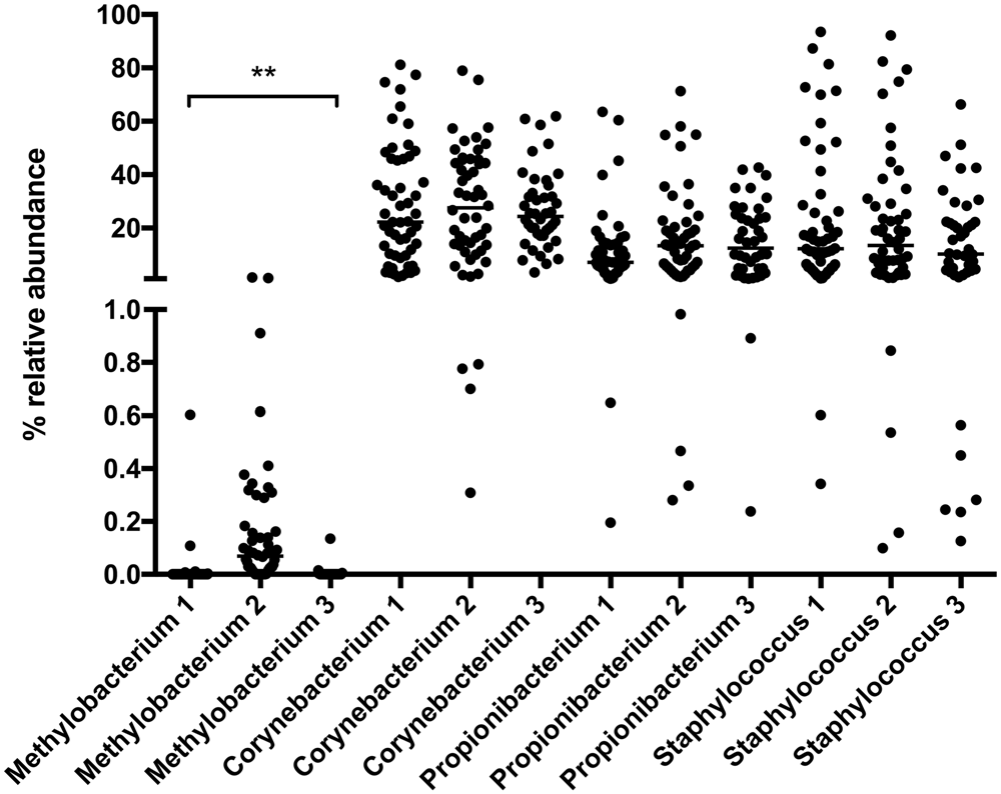
Relative abundance of selected genera in anterior nasal microbial communities according to swab type. Shown are those genera that either were the most abundant or showed a statistically different abundance across swab types (indicated as 1, 2 and 3, respectively; see Methods for details about the swabs). **p < 0.01 (Kruskall-Wallis test with post-test Dunn’s). The horizontal lines indicate median values.

The abundances of genera detected with each of the three swab types were compared using the Kruskal-Wallis test on standardized abundance data of 148 anterior nares and 110 oropharyngeal samples obtained from 258 participants. In anterior nares, out of the 95 detected genera only *Methylobacterium* spp. differed in abundance across swab types in that it was more frequently detected with swab 2 (p = 0.0077, Fig. 6). In oropharynx all genera were detected with equal frequency by all three swab types.

#### Comparison of staff-collection vs. self-collection at home of anterior nasal swabs

**Figure 7:**
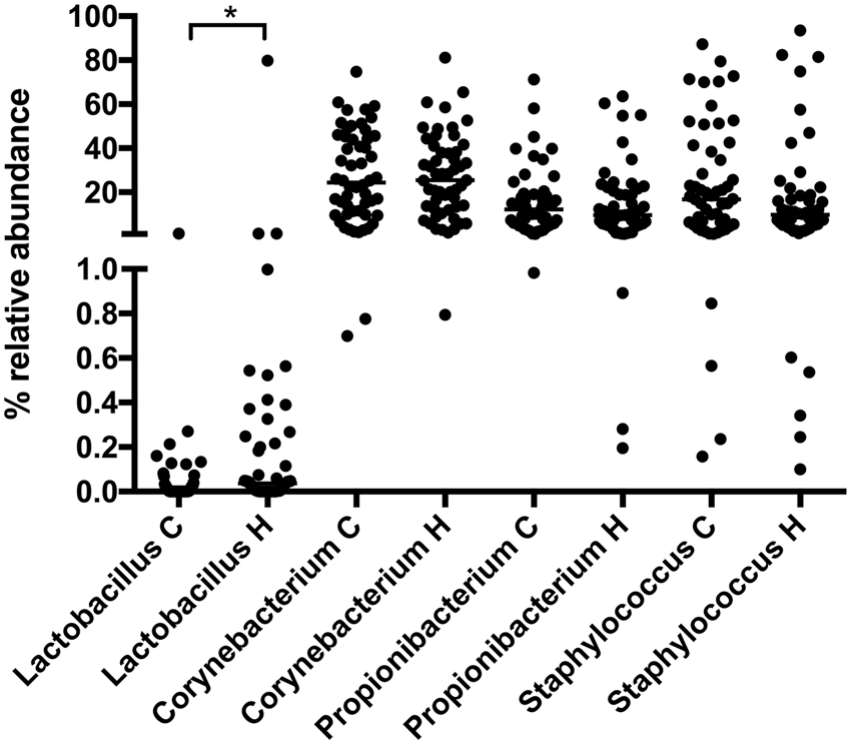
Relative abundance of selected genera in nasal microbial communities. The method of swab collection is indicated on the x-axis: collected in the study center = C; collected at home = H. Only samples that had a perfect match in participant age and sex were considered (54 samples each). Statistically significant differences in genus abundances between samples taken at home by study participants (H) and by certified study personnel at the study center (C) were calculated by the Mann Whitney U test, *p < 0.05; ***p < 0.001. Median values are indicated by the horizontal lines.

We then tested whether, for the purpose of microbiome determination, nasal self-swabbing at home is as suitable as swabbing by the certified personnel in the study centers. The communities of 54 nasal swabs self-collected by the participants at home were compared to 54 age and sex matched staff-collected swabs taken at the study centers. As observed above in the comparison of the three swab types, there were no differences in the overall communities (ANOSIM: global R = 0.019, PERMANOVA: Pseudo-F = 1.909, P = 0.061) nor were there any differences in diversity indices between the self-collected and the staff-collected swabs. Only members of the genus *Lactobacillus* were distributed differentially (p = 0.0222; Fig. 7).

## Discussion

This is the first study that formally examined acceptance and feasibility of nasal and oropharyngeal swabbing in a multicenter population-based epidemiological study, and coupled this with detailed characterizations of microbial communities in these two ecological niches of great importance to human health.

### Feasibility of integrating anterior nasal microbiome analyses into population-based studies

As expected, acceptance of nasal swabbing by both study personnel and study participants was very high in all study centers, and the short time required for swab collection and processing is well within the range that can be easily integrated into large human studies. Of note, our results also clearly show the equivalence of staff-collected and self-collected nasal swabs. In particular, the data demonstrate the robustness of microbiome determinations from nasal swabs to differences in temperature after collection when RNAlater is used as a preservative: whereas the staff-collected swabs were frozen shortly after collection, the self-collected swabs were not frozen until receipt in the study center (usually 1 or 2 days after collection). The observed high feasibility of nasal self-swabbing at home agrees well with previous results obtained in somewhat different settings, where self-collection of nasal swabs was found to be feasible for capturing viral pathogens causing acute respiratory infections^8–11^ and for the serial determination of *S. aureus* colonization in a population-based setting^2^. Self-swabbing is a highly cost-effective method, particularly when serial swabs are to be collected, which would normally require multiple visits to the study center. Our data indicate that it can be used as an alternative to staff-collection in population-based studies featuring NGS-based analyses of anterior nasal microbiomes (next-generation sequencing).

### Particular aspects of oropharyngeal swabbing

There is a common opinion that oropharyngeal swabbing might have negative effects on acceptance and compliance in prospective population-based studies due to the difficulties of the swabbing procedure in this anatomical location, and that this may result in poor response rates and/or high attrition rates. The observed high acceptance of oropharyngeal swabbing by the participants and their stated willingness to participate in future studies featuring oropharyngeal swabbing suggest that this may not be the case, at least if swabbing is performed by trained personnel (as was the case in our study). Nonetheless, despite the training provided to the study personnel before study start, completeness of oropharyngeal swabbing (as measured by the fraction of the anatomical structures swabbed successfully) varied among the study centers, and the degree of participants’ discomfort was clearly higher than in the case of nasal swabbing. Thus, particular attention should be paid to training study personnel in technique that aims to maximize completeness of swabbing of oropharyngeal structures, while minimizing discomfort.

### Microbiological validation

The microbial community structure analyses largely agree with previous studies of anterior nasal and oropharyngeal microbiomes, thus underscoring the microbiological validity of our approach. Of note, despite the difficulties encountered with oropharyngeal swabbing discussed above, representative microbial communities were detected with nearly all oropharyngeal swabs, underscoring the analytical robustness of this method. The swab comparison showed that all three swab types included in the laboratory analyses are suited equally well for determination of nasal and oropharyngeal microbiomes in population-based settings. This is particularly important because the choice of swab may vary among future studies in other locations; our data suggest that comparability of data should be good if one of these three swab types is used.

### Limitation of the study

Due to the design of the Pretest 1 studies across all study centers, it was not possible to apply culture-dependent microbiological methods at the time of swabbing, and we therefore do not have data from this classical approach about the presence of lead organisms such as *Streptococcus spp*. and *Staphylococcus spp*. in parallel with the NGS-based microbiome data.

### Conclusions

Nasal and oropharyngeal swabbing turned out to be highly accepted and feasible methods to collect NGS-based data on bacterial microbiomes in these two ecological niches in population-based studies. They can thus be used in larger-scale population-based studies on nasal and oropharyngeal microbiomes and their effects on health and disease of the host.

## Methods

### Study population

The study was integrated into the Pretest 1 phase of the GNC, which was conducted in 2011 and aimed to test the feasibility of a variety of aspects of recruitment, assessments and examinations in the study center, and follow-up^12–14^. All study centers drew random population-based samples from the respective residents’ registration offices. In brief, invitations were sent out by mail and included a description of the aims and examination procedures of the study and a copy of the approval letter from the data protection commissioner. A reminder letter was sent if there was no response after four weeks. Individuals were additionally contacted by phone, if a phone number was available. Out of the 17 centers participating in Pretest 1, the feasibility of obtaining nasal and oropharyngeal swabs was tested in six and four study centers, respectively. Three of these study centers also examined the feasibility of obtaining self-collected nasal swabs at home. Fig. 1 shows the geographical locations of the six study centers and the type of swabbing conducted at each center. All participants in Pretest 1, and thus the presented feasibility study, also participated in a basic face-to-face interview to obtain commonly assessed demographic, socioeconomic and medical data, in anthropometric measurements (height and weight), heart rate and blood pressure measurements, the collection of other biosamples, and in other feasibility studies.

### Nasal and oropharyngeal swabbing

#### Training of study personnel

The local study teams consisted of 2-4 individuals (both physicians and study nurses) and were trained and certified on site by the coordinator of the presented study, who is a licensed physician and has extensive personal experience in obtaining nasal and pharyngeal swabs according to internationally accepted Standard Operating Procedures (SOPs). Training sessions consisted of three parts: (a) a Powerpoint presentation (reviewing the aims of the study, relevant anatomy, sampling technique and sample preservation), (b) demonstration and hands-on training, and (c) certification (each trainee had to demonstrate three correctly executed swabs).

#### Swabbing procedure

In the study center, the certified personnel explained the study aims to the participant and then collected from him/her one anterior nasal swab from each nostril and one oropharyngeal swab. For nasal swabbing, the swab was inserted into the nostril to a depth of 1–1.5 cm and rotated three times on the nasal lining under constant pressure. This procedure was repeated for the other nostril. Both swabs were then placed into one 1.5 ml storage tube (Micro tube, Sarstedt, no. 72.694.005) containing 1 ml RNAlater® Stabilization Reagent (QIAGEN, Austin, Texas, USA). Furthermore, the study personnel swiped the oropharyngeal swab over the tonsils (or “tonsillar areas” if tonsils were atrophied or had been removed) and pharyngopalatine arches bilaterally. The fraction of these anatomical structures that was swabbed successfully was recorded in the biosample protocol and was used as a proxy for completeness of the pharyngeal swabbing procedure. After completing the swabbing procedure, the swab tip was clipped off and placed in a 1.5 ml storage tube containing 1 ml RNAlater^®^. After collection, the swabs were placed on dry ice and transferred to −80 °C storage by the end of the day.

Lastly, in three study centers (Fig. 1), the participants were instructed how to self-collect nasal swabs. Four weeks later, a swabbing kit containing two nasal swabs, a transport tube containing 2 ml RNAlater, a small bottle containing 2 ml NaCl, a questionnaire, instructions how to self-collect a nasal swab, and gloves were sent to the participants. They were asked to self-collect one nasal swab from each nostril, complete a questionnaire and mail everything to the laboratory within 24 h. The swabbing procedure at home was described in a manner similar to that in the study center. In brief, the nasal swab was to be inserted into the nostril to a depth of approx. 1.5 cm, rotated three times on the nasal lining under constant pressure and placed into RNAlater. The participants were instructed to keep the swabs in the refrigerator overnight if they were collected the day before shipping. The swabs were shipped at ambient temperature and placed at −80 °C upon arrival in the study center.

#### Swab types

The following four swab types were compared: swab 1–flocked nylon swab (Nr. 502CS01, Hain Lifescience), swab 2–rayon swab (Nr. 30MW112, Check Diagnostics GmbH), swab 3–polyurethane tipped swab (Nr. 30MW940/125, Check Diagnostics GmbH), and swab 4–calcium alginate tipped swab (Nr. 25-806 1 Pa, Check Diagnostics GmbH). The study participants were randomized by block randomization to one of the four swab types. In a given participant, the same swab type was used for nasal and oropharyngeal swabbing and for nasal self-swabbing.

### Acceptance

After completing the swabbing procedure, the participants were asked to fill in separate acceptance questionnaires for nasal and oropharyngeal swabbing at the study center and nasal self-swabbing at home. Acceptance was assessed with questions on a five-point Likert scale. Participants rated each item with 1 (strong disagreement), 2 (disagreement), 3 (neither agreement nor disagreement), 4 (agreement), or 5 (strong agreement). Some of the items were reverse-phrased to reduce response bias.

### Laboratory analysis

Out of the 1807 swabs collected, a subsample of 313 swabs (195 nasal and 118 oropharyngeal swabs, originating from 258 participants) was selected for microbiome analysis, as this number was deemed large enough to achieve the major goals of the study (Table 3, supplemental Table 3). To avoid any possible bias introduced by incorporating multiple swabs from the same participant, only 258 swabs (148 samples from the anterior nares and 110 oropharyngeal samples, see Table 3 and supplemental Table 3) were analyzed for comparing swab types or self-swabbing vs. swabbing by trained personal. Separate swabs were collected from each nostril, but only one of the two swabs was randomly taken for analysis, resulting in a random mixture of swabs from the left or the right nostril for subsequent analyses.

**Table 3:** Overview of swabs included in the microbiome analysis.

	Nasal swabs collected by certified personnel at the study center			Oropharyngeal swabs collected by certified personnel at the study center			Nasal swabs collected by study participants at home		
Swab type	swab1*	swab2**	swab3***	swab1*	swab2**	swab3***	swab1*	swab2**	swab3***
No. of swabs	23	26	25	39	34	37	31	26	17
Total no. of swabs analyzed	74			110			74		

313 samples originating from 258 individuals were analyzed. To avoid possible bias, a subset of analyses was performed on 258 samples comprising only one swab per individual.

*Swab 1 – flocked nylon swab.

**Swab 2 – rayon swab.

***Swab 3 – polyurethane tipped swab.

Swab 4 (calcium alginate) was excluded because it had proven unsuitable during preanalytical processing (see Results).

Swabs were placed into tubes containing 500 µl nuclease-free H_2_O and 100 mg of glass beads (acid-washed, ≤ 106 µm, Sigma-Aldrich). Cells were lysed in a FastPrep^**®**^-24 Instrument for 30 seconds at an intensity setting of 6. DNA extraction was performed using the InstaGene Matrix (Biorad) following the manufacturer’s instructions. Amplicon libraries were generated of the V1–V2 region of the 16 S rRNA gene using the primers 27 F and 338R^6^ in a 20 cycle PCR reaction as previously described^8^. Amplicons were extracted from agarose gels with the help of Qiagen’s QIAquick Gel Extraction Kit according to the manufacturer’s protocol. Before sequencing, the concentration of the cleaned-up PCR products was quantified with the Quant-iT PicoGreen dsDNA Kit (Invitrogen) and samples were pooled in equal concentrations. Sequencing of 2 × 250 bp was performed on an Illumina MiSeq Sequencer (Illumina, Hayward, Californa, USA). Raw sequences were merged^15^ and subsequently aligned using MOTHUR (gotoh algorithm with the SILVA reference database) prior to pre-clustering, allowing two mismatches. A total of 14.5 million sequences for 313 samples (46,399 ± 28,247 sequences per sample) were analyzed. Only phylotypes with an average abundance of at least 0.001% of the total communities and a sequence length of >200 bp were considered for follow-up analysis. All phylotypes were assigned a taxonomic affiliation based on naïve Bayesian classification (RDP classifier)^16^. Phylotypes were then manually analyzed against the RDP database using the Seqmatch function as well as against the NCBI database to define the discriminatory power of each sequence. Sequences not originating from 16 S rDNA were deleted, leaving a total of 13.2 million sequences. The minimal number of reads per sample was 8850. A species name was assigned to a phylotype when only 16 S rRNA gene fragments of previously described isolates of that species showed ≤2 mismatches with the respective representative sequence read. Similarly, a genus name was assigned to a phylotype when only 16 S rRNA gene fragments of previously described isolates belonging to that genus and of 16 S rRNA gene fragments originating from uncultured representatives of that genus showed ≤ 2 mismatches. Relative abundances of bacterial phyla or phylotypes were then determined by summing up all sequences and calculating the relative share of each phylum/phylotype to the whole microbial community.

### Statistical analysis

All analyses were computed on standardized abundance data. Sample-resemblance matrices were calculated on the basis of the Bray-Curtis algorithm^17^. Bacterial community structures of *a priori* defined groups were visualized by ordination using nMDS with 50 random restarts. Significant differences between a priori predefined groups of samples were evaluated using Analysis of Similarity (ANOSIM) (9999 permutations) with the accompanying R statistic measuring the degree of separation between groups^18^ and/or permutational multivariate analysis of variance (PERMANOVA), allowing for type III (partial) sums of squares with a fixed effects sum to zero for mixed terms and exact p-values generated using unrestricted permutation of raw data^19^. Groups of samples were considered significantly different if the p-value was <0.05. The degree of intra-variation for samples from the same habitat was calculated by applying multivariate dispersion analysis, where a low dispersion index indicates low within-group heterogeneity. Phylotype richness, diversity, and evenness were explored by calculating total phylotypes (S), Simpson’s Diversity Index (1-D), Shannon Diversity Index (H′) and Pielou’s Evenness Index (J′) on subsampled data using the function *rarefy_even_depth (rngseed* = *TRUE)* from the R package “phyloseq”^20^.

The abundances of species-of-interest and univariate diversity indices were compared between *a priori* groups of samples using either a t-test or analysis of variance (ANOVA). Species abundance data and diversity indices were subjected to a normality test using both the D’Agostino & Pearson omnibus and the Shapiro-Wilk algorithms (Prism 6, Graphpad Software Inc). Since most of the PT abundances across most of the groups returned estimates indicating non-normal distributions, the Kruskal-Wallis test with Dunn’s multiple comparison post-hoc test was used when multiple groups were being compared, whereas the Mann-Whitney test was used when only 2 groups were compared. The Benjamini-Hochberg correction was applied for multiple comparisons^21^. Groups of samples were considered significantly different if the *p*-value was <0.05. The multivariate analyses were performed and diversity indices were calculated using PRIMER (v7.0.6; PRIMER-E; Plymouth Marine Laboratory, UK)^18^ whereas the univariate analyses and normality tests were performed in Prism 6 (Graphpad Software, Inc.).

### Ethics approval

All procedures performed in studies involving human participants were in accordance with the ethical standards of the institutional and/or national research committee and with the 1964 Helsinki declaration and its later amendments or comparable ethical standards. The study was approved by the responsible ethics committees of the German Federal States in Augsburg, Bremen, Essen, Freiburg, Hamburg and Heidelberg. Informed consent was obtained from all individual participants included in the study.

## Electronic supplementary material

Dataset 1
